# ECONOMIC IMPACT OF COVID-19

**DOI:** 10.1590/1413-785220233101e254289

**Published:** 2023-02-20

**Authors:** RUJITTIKA MUNGMUNPUNTIPANTIP, VIROJ WIWANITKIT

**Affiliations:** 1 Private Academic Consultant, Bangkok, Thailand.; 2 Dr D. Y. Patil Vidyapeeth University, Pune, MH, India.

To the Editor, we would like to share ideas on the publication “Economic impact of COVID-19 on Brazilian orthopedists.”^1^ Stirma et al. concluded that “There was a direct relationship between the professional experience in this subspecialty and a higher percentage of fixed income, as well as a greater impact on the reduction percentage in the monthly budget and a longer time off the job.”^1^ The pandemic affected many people worldwide, including medical personnel. People working at a private establishment were probably affected by the lockdown. Some kinds of clinic, especially surgery clinics, had to be closed during COVID-19. The lockdowns may have significantly increased workload. In this period, more than 15% loss of income was reported.^2^

In developing countries, the use of new technology might help solve the problem. A recent report from USA showed that orthopedic surgeon still maintain income via teleconsultant.^3^ However, this might be impossible in many settings, such as our setting in Asia, where the infrastructure is poor and the patient cannot access the telemedicine system. The negative effect of lockdown on orthopedic surgery and decrease of income is a common problem.
